# A 100 Gb s^−1^ quantum-confined Stark effect modulator monolithically integrated with silicon nitride on Si

**DOI:** 10.1038/s44172-025-00421-6

**Published:** 2025-05-01

**Authors:** Ilias Skandalos, Thalía Domínguez Bucio, Lorenzo Mastronardi, Guomin Yu, Aaron Zilkie, Frederic Y. Gardes

**Affiliations:** 1 Optoelectronics Research Centre, University of Southampton, Southampton, UK; 2 Rockley Photonics Inc., Pasadena, CA USA

**Keywords:** Integrated optics, Technology, Photonic devices

## Abstract

The exponential growth of data-intensive artificial intelligence necessitates ultra-fast and energy efficient transceivers in data centres. Quantum-confined Stark effect (QCSE) modulators offer promising solutions, combining high-speed modulation with minimal footprint and superior energy efficiency. Here, we demonstrate a monolithically integrated O-band Ge/SiGe QCSE modulator operating at 100 Gb s^−1^, seamlessly integrated with silicon nitride (SiN) waveguides on both silicon and silicon-on-insulator substrates. Our modulator achieves <1 dB coupling loss,  <63 fJ bit^−1^ energy consumption, and  >5 dB static extinction ratio, while maintaining performance across a 20–80 °C temperature range. Leveraging CMOS-compatible fabrication processes, we incorporate multiple quantum-well stacks grown at wafer scale on silicon, enabling large-scale production. The modulator’s substrate-agnostic integration with back-end of line grown SiN layers, presents a scalable approach for cost-effective co-integration of electronic and photonic components. This work advances high-speed, energy-efficient optical modulators and paves the way for next-generation photonic integrated circuits in data centre interconnects.

## Abstract (web summary)

Ilias Skandalos and colleagues demonstrate a silicon-germanium quantum-confined Stark effect modulator on 8-in. silicon wafers, monolithically integrated with silicon nitride waveguides under sub-dB coupling losses. The modulator achieves a 100 Gb s^−1^ data rate and a >65 GHz bandwidth with <63 fJ bit^−1^ energy consumption.

## Introduction

The rapid escalation in global data traffic, driven by advancements in artificial intelligence (AI)^1^ and the widespread adoption of 5G technology^2^, is placing unprecedented pressure on existing data centre infrastructure. Consequently, there is an increasing demand for advanced transceivers, particularly those exceeding 800G capacities, which need to be also integrated into a space-efficient, co-packaged electronic-photonic environment^3^. Central to this integration is the development of high-speed photonic components, capable of operating at least at 100 Gb s^−1^ in on-off keying (OOK) mode, while maintaining a compact physical footprint (<150 μm^2^) and an optimal energy efficiency (<100 fJ bit^−1^)^4^. Fabrication on a complementary metal-oxide semiconductor (CMOS) substrate is essential due to its maturity in mass production and its versatility in supporting various materials for a wide array of applications. Silicon photonics stands out as a promising solution for these future photonic integrated circuits^5^, benefiting from the well-established CMOS manufacturing processes and its compatibility with a diverse material integration^6^.

The semiconductor optical sources for datacentres operating in the O-band (1260 nm–1360 nm), typically based on III/V materials^7^, are either directly or externally modulated. External modulation offers advantages such as higher radio frequency (RF) link power gain (G) and lower noise figure (NF) compared to the direct modulation, taking advantage of the proportionality between the link gain and the square of the input optical power (*P*_*I*_) to the modulator ($G\propto {{P}_{I}}^{2}$ and $NF\propto 1/G\propto 1/{{P}_{I}}^{2}$). In contrast, direct modulation exhibits no such dependency between *G* and *P*_*I*_, as demonstrated in the works of Cox et al. in refs. ^8,9^. A mature external option is the Mach-Zehnder Interferometer (MZI) modulator, capable of achieving high-speed response (112 gigabaud^10^) coming though with a high power consumption and a large footprint. On the contrary, electro-absorption modulators (EAM) based on CMOS-compatible bulk structures^11^ or multiple quantum-well (MQW) stacks^12^ offer a significantly smaller footprint (<150 μm^2^) providing a pathway to all band high density integration of stand alone optical modulators. Monolithic C-band (1530 nm–1565 nm) short (<50 μm) and energy efficient (<50 fJ bit^−1^) Franz-Keldysh Si/SiGe modulators^11^, along with compact silicon micro-ring modulators showing speeds of  >100 Gb s^−1^^13^ have been demonstrated. Nevertheless, an equivalent monolithic CMOS-compatible O-band component, which utilises efficient waveguide-integrated and high data rate QCSE modulation, has yet to be realised.

Material strain-engineering in stratified media of quantum well and barrier layers, based on semiconductor alloys on silicon (Si) substrates, can enable the O-band modulation by exploiting the QCSE. QCSE modulators have been studied in p-i-n diode forms by using III/V^14–17^ and silicon germanium (Si-Ge)^18–21^ stacks, with robust epitaxial growth techniques for the latter due to its structural similarities with Si. However, even for the more mature Si-Ge stack a waveguide integration is challenging. An evanescent coupling format using a Si-Ge taper is difficult to control during fabrication^18^, whilst a butt-coupling connection is limited by the discrepancy in the core thickness of the passive and the active parts (220 nm in standard silicon-on-insulator (SOI)^5^,  ~400 nm–1000 nm in Si-Ge^22^). Furthermore, achieving uniform growth of multiple MQW layers within SOI wafer trenches, while ensuring smooth interfaces to the passive sections, presents a number of significant challenges. Specifically, the grown layers of the MQW stack inside the trenches tend to suffer non-uniformity near the trench edges, resulting in an unevenness of their thickness inside the etched area. This non-uniformity complicates the precise alignment of the active waveguide relative to the passive waveguide, increasing errors and degrading coupling performance due to increased coupling losses (CL) and back reflections (BR). Moreover, an uneven stack can result to a non-uniform electric field along the modulator with unpredictable responses near the interfaces, leading to reliability and repeatability issues of the devices.

The integration difficulties reported above can be overcome by implementing the coupling strategy described in ref. ^23^ by Skandalos et al., which involves the growth of silicon nitride (SiN) waveguides (instead of the MQW stack) in the trenches of a wafer with the blanket active stack (instead of a wafer with the passive waveguide) targeting a butt-coupling connection. Specifically, plasma-enhanced chemical vapour deposition (PECVD) SiN^24^ can be deposited in trenches of a Ge/SiGe MQW stack wafer without affecting the doped regions due to its low-temperature (<350 °C) growth conditions. Furthermore, its amorphous structure allows a defect-free deposition, which is adaptive to the thickness of the MQW stack. In that manner, the wafer-scale uniformity of the MQW stack thickness is guaranteed with no physical distortions at the waveguide interfaces, ensuring a reliable modulation performance. Also, the adaptivity of the SiN to the MQW stack enables a better mode matching between the two waveguides, minimising unwanted parasitic back-reflections. Furthermore, the versatility of the SiN compositions from nitrogen-rich (N-rich SiN, *n* < 2) to stoichiometric (Si_3_N_4_, *n* = 2) and silicon-rich (Si-rich SiN, *n* > 2) can provide a wide refractive index range from 1.7 up to 2.9, allowing the fabrication of index-matching anti-reflective coating (ARC) layers.

Consequently, a robust monolithic active-to-passive transition can be realised through the use of silicon nitride, which can also be utilised as the basis of the passive circuitry^25^. Owing to its low thermo-optic coefficient (~10^−5^ °C^−1^), the temperature variation impact on the propagating mode is minimised. Furthermore, silicon nitride is comparatively more resilient against fabrication uncertainties due to its moderately confined propagating modes (mid-index, *n* ~ 2), which in combination with the temperature insensitivity makes it attractive for wavelength division (de)multiplexing (WDM) applications^26,27^.

The integration strategy described above can be undertaken using either Si or SOI substrates. Specifically, the MQW stack can be grown on silicon either in the form of a thick substrate or a standard 220 nm SOI epilayer, followed by the low-temperature PECVD SiN. In the SOI case, silicon nitride can act as the bridging pathway to the SOI waveguides via evanescent coupling and adiabatic tapers (<0.1 dB/transition^28,29^), enabling a flexible photonic integration with varying applications (e.g. temperature insensitive (de)multiplexers in SiN and tight bendings in SOI), taking advantage of the best properties of both platforms. It is noted that under this approach, a variation in the thickness of the top surface of the SOI epilayer is expected, nevertheless, this can be minimised by using selective etching processes of SiGe against Si, resulting in a smooth silicon substrate surface after the SiGe layers' removal. Moreover, the Si-substrate scenario is aligned with a low-cost CMOS electronic-photonic large-scale integration^30^, while a MQW growth without an insulating oxide beneath avoids self-heating issues for the electronic components^31^ and the photonic modulator response^32^, enhancing further the device reliability.

**Fig. 1:**
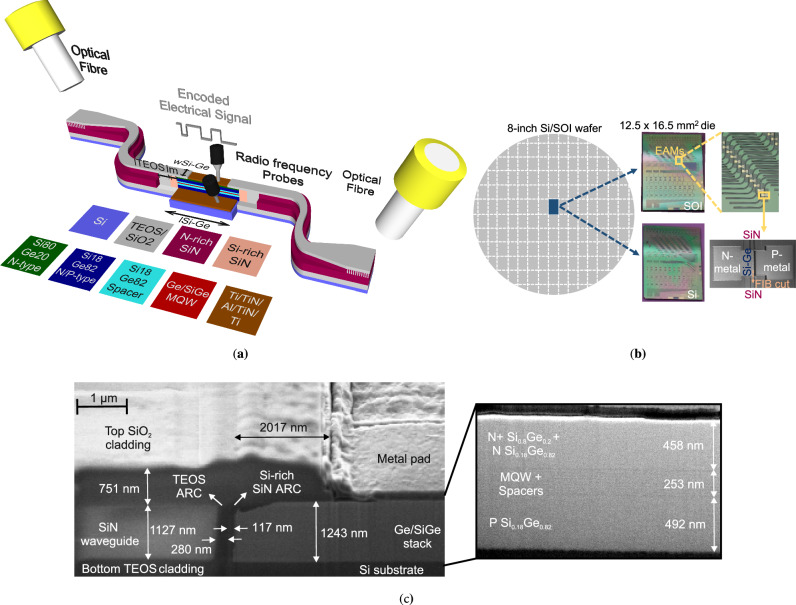
The QCSE modulator integrated with SiN waveguides. **a** Conceptualisation of the SiN-integrated Ge/SiGe MQW electro-absorption modulator on a Si substrate. Optical power is sent and received via optical fibres and straight grating couplers on N-rich SiN waveguides. A coupling-efficient transition between the N-rich SiN and the Ge/SiGe waveguides is achieved through a double-layer TEOS/Si-rich SiN anti-reflective coating. Metallic pads are placed on top of the N- and P-type contact layers of the modulator. The modulator is reverse-biased through radio frequency probes for static and high-speed QCSE operating modes. **b** The SiN-integrated QCSE modulator was demonstrated on 8-in. Si and SOI wafers, that were diced into 12.5 × 16.5 mm^2^ dies. An example of the fabricated and characterised modulators can be seen through microscope and scanning electron microscope (SEM) images. **c** Scanning electron microscope images of the SiN/Si-Ge interface on a Si wafer.

In this work, we propose and demonstrate a new novel approach to monolithically integrated O-band Ge/SiGe EAMs with silicon nitride waveguides on both Si and SOI substrates. A visual representation of the electro-optic operation concept of the EAM on a Si substrate is shown in Fig. 1a. The waveguide-integrated modulators are fabricated on 8-in. <100> Si and SOI wafers via deep-ultraviolet lithography (DUV) and further diced into 12.5 × 16.5 mm^2^ dies as seen in Fig. 1b.

A scanning electron microscope (SEM) image of the interface, which was cut via a focused ion beam (FIB), is shown in Fig. 1c. The FIB cut direction is defined in Fig. 1b with the small dashed arrow. The distinct areas of the N-rich SiN, TEOS (ARC layer 1), Si-rich SiN (ARC layer 2), and Si-Ge are shown, along with their related geometric parameters. Also, the SiO_2_/TEOS claddings, the Si substrate and the metal pad along with its recess area, are depicted. The interface appears smooth without defects. Due to the very small thickness (~10 nm) of the MQW layers, the MQW region can be seen in the more MQW-focused SEM image in Fig. 1c. Details about the interface design and the waveguide coupling performance can be found in Supplementary Fig. 1, Supplementary Table 1, and Supplementary Fig. 2, respectively.

## Results

### Static performance

**Fig. 2:**
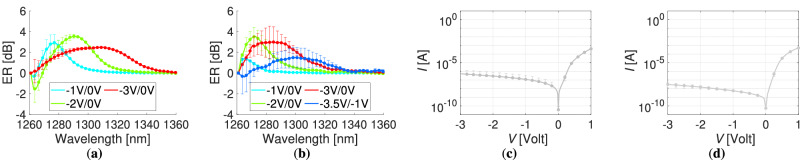
Static performance of the modulator. Extinction ratio for different reverse bias values in reference with 0 V for the (**a**) Si and the (**b**) SOI substrates. The −3.5 V/−1 V curve is also shown for the SOI case. The corresponding I-V characteristic curves are plotted in (**c**) and (**d**) for the Si and SOI cases, respectively. The data are shown in the form of mean values accompanied by the standard deviation of the measurements for three central chips on the wafer.

The static electro-optic performance of the modulator was assessed in the O-band. For different bias voltages between  −3 and  −1 V the extinction ratio (ER) calculated with reference to the 0 V case is plotted in Fig. 2(a) and (b) for the Si and SOI cases, respectively. The measurements refer to a 50 μm long and 2.5 μm wide Si-Ge section in three different chips at the centre of each wafer covering a circular area of  ~2 cm radius. The measurement results are represented graphically, with the mean values indicated by dots and the standard deviation displayed as error bars. Within this range of reverse bias, the exciton peaks in the absorption coefficient spectrum undergo a red shift due to the QCSE, achieving their maximum value at a distinct wavelength for each set of reverse bias values^33^.

The observed shift is reflected in the ER values, which are calculated as *ER*_dB_ = $10{\log }_{10}\,\Big(\frac{I{L}_{{V}_{a}}}{I{L}_{{V}_{ref}}}\Big)$, where $I{L}_{{V}_{a}}$ and $I{L}_{{V}_{ref}}$ represent the insertion loss (IL) levels under the applied reverse bias *V*_*a*_ and the reference reverse bias *V*_*ref*_, respectively. The ER denotes the ratio of extinction between these two states, with the insertion losses as measured via grating-coupled direct current (DC) measurements. In this method, all other sources of loss are normalised, with the exception of the absorption due to the QCSE. The associated current-voltage (I-V) characteristic curves for both Si and SOI wafers are depicted in Fig. 2c, d, respectively, using a semi-logarithmic scale. In the absence of illumination, the dark current remains below 0.53 ± 0.6 μA for reverse biases up to −3 V on the Si substrate and below 0.03 ± 0.017 μA for the SOI substrate. Under forward bias, the current increases linearly, exhibiting a typical diode behaviour. In the case of the Si substrate, the highest ER of 3.55 ± 0.22 dB is observed at 1290 nm for the  −2/0 V bias pair. This peak ER value shifts, diminishes, and becomes more broadly distributed spectrally under a  −3 V bias. A similar trend is noted for the SOI substrate, but the most pronounced ER peak occurs at a shorter wavelength of 1272 nm, reaching 3.5 ± 1.27 dB for the  −2/0 V pair. As the reverse bias is increased, this peak undergoes a red shift and attains a value of 2.8 ± 1.59 dB for the  −3/−1 V pair.

**Fig. 3:**
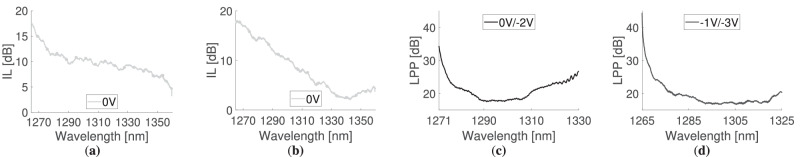
Loss performance of the modulator. Insertion loss of the modulator at 0 V for the (**a**) Si and the (**b**) SOI substrates. The corresponding link power penalty curves are shown in (**c**) and (**d**) for the Si and SOI cases, respectively.

The insertion loss at 0 V for both Si and SOI substrates is depicted in Fig. 3a, b, respectively. This loss encompasses the total loss in the Si-Ge section (including Ge and SiGe indirect absorption, doped Si absorption, Si-rich SiN absorption, losses due to metal pads, and scattering loss) as well as two SiN/Si-Ge interfaces. Incremental variations in interface geometric parameters were implemented through lithographic resist exposure and dosage adjustments (see Supplementary Information for more details). Consequently, the lowest insertion loss was attained in a specific area of the wafer, and the minimum measured insertion loss is presented here without statistical variation. For the Si substrate, the optimal insertion loss at 1290 nm is recorded as 9.57 dB at 0 V. For the SOI substrate, the best-case scenario insertion loss at 1272 nm, 1290 nm, and 1300 nm is 16.88 dB, 12.09 dB, and 10.31 dB, respectively. The extinction ratio and insertion loss metrics can be combined to calculate the link power penalty (LPP), defined as $10{\log }_{10}(2{P}_{{{{\rm{in}}}}}/({P}_{{{{\rm{1}}}}}-{P}_{{{{\rm{0}}}}}))$, where *P*_in_ is the input optical power to the modulator (just before the SiN/Si-Ge interface), and *P*_0_, *P*_1_ represent the optical power levels for the ‘OFF’ (*V*_a_ reverse bias) and ‘ON’ (*V*_ref_ reference reverse bias) states, respectively. The lowest LPP for the Si substrate under the  −2/0 V bias pair is 17.5 dB at 1291 nm. In the case of the SOI substrate and for the  −3/−1 V bias pair we observe a slightly lower extinction ratio compared to the  −2/0 V pair. Furthermore the insertion loss is significantly lower at the corresponding peak wavelength (12.1 dB at 1290 nm and 8.95 dB at 1305 nm when biased at  −1 V). Thus, the optimal LPP value is calculated to be 16.66 dB at 1305.4 nm, while at 1290 nm, the penalty is 17.69 dB.

### High-speed performance

**Fig. 4:**
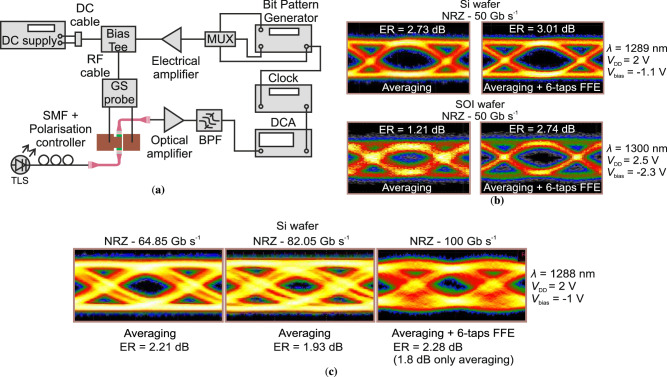
High-speed operation of the modulator. **a** Characterisation setup. Eye diagrams for an NRZ modulation at 50 Gb s^−1^ in the case of (**b**) the Si wafer and the SOI wafer. **c** Higher data rate eye diagrams for a Si-substrate device and for NRZ modulation at 64.85 Gb s^−1^, 82.05 Gb s^−1^ and 100 Gb s^−1^. DC direct current, RF radio frequency, MUX multiplexer, GS ground-signal, SMF single-mode fibre, TLS tunable laser source, BPF bandpass filter, DCA digital communication analyzer.

The excitonic resonance occurring in the QCSE is a sub-picosecond process, as described by Schmitt-Rink et al.^34^. This indicates that the high-speed capabilities of the EAM are primarily governed by the RC constraints of its equivalent circuit. To explore the upper bounds of the EAM’s high-speed transmission rates, eye-diagram tests were conducted under a non-return-to-zero (NRZ) OOK operation. The setup employed for these eye-diagram measurements is illustrated in Fig. 4a.

The high-speed transmission performance of the EAM on both substrates was initially verified at 50 Gb s^−1^, as depicted in Fig. 4b. The initial seed signal, at 50 Gb s^−1^ from an SHF multiplexer (MUX), had an amplitude of ~500 mV. This signal, after traversing a 0.5 m long RF cable, was amplified by a 60 GHz amplifier to achieve the necessary voltage swing. Subsequently, the signal was routed through a 65 GHz bias tee and a 50 Ω-terminated probe to activate the EAM. Different operational regimes produced the optical-eye diagrams shown in Fig. 4b. Specifically, the device on the Si substrate was operated at 1289 nm, with a bias of  −1.1 V and a 2 V voltage swing, following the optimal ER region shown in Fig. 2a. Applying an averaging function on the received optical signal yielded an ER of 2.73 dB. The optical eye was further opened using a 6-tap feedforward error (FFE) correction, which compensated for the relatively high noise level in the DCA signal that had a  ~8 signal-to-noise ratio (SNR), resulting in an ER of 3.01 dB. For the SOI-substrate device, the ER values were 1.21 dB with averaging and 2.74 dB with the addition of 6-tap FFE. This modulator operates at 1300 nm, with a 2.5 V voltage swing and a bias of  −2.3 V, consistent with the findings in Fig. 2b (−3.5 V/−1 V curve). The lower ER values compared to the Si-substrate modulator are attributed to a higher noise level (SNR  ~2.9), which, as a measurement artefact, affects the characterised ER level. This is further corroborated by the improved ER of the SOI-substrate when employing 6-tap FFE, reaching values nearly equal to those of the Si-substrate modulator.

The high speed limits of the modulator were further investigated, by operating the modulator under the data rates of 64.85 Gb s^−1^, 82.05 Gb s^−1^ and 100 Gb s^−1^ and achieving the eye diagrams of Fig. 4c. This study refers to the Si-substrate device as the SNR was very low to achieve an open eye diagram for the SOI-substrate modulator. The corresponding ER values obtained were 2.21 dB, 1.93  dB, and 1.8 dB, respectively. At the speed of 100 Gb s^−1^ a 6-tap FFE was added, reaching an ER level of 2.28 dB. The modulator was biased at −1 V with a voltage swing of 2 V and operated at 1288 nm.

**Fig. 5:**
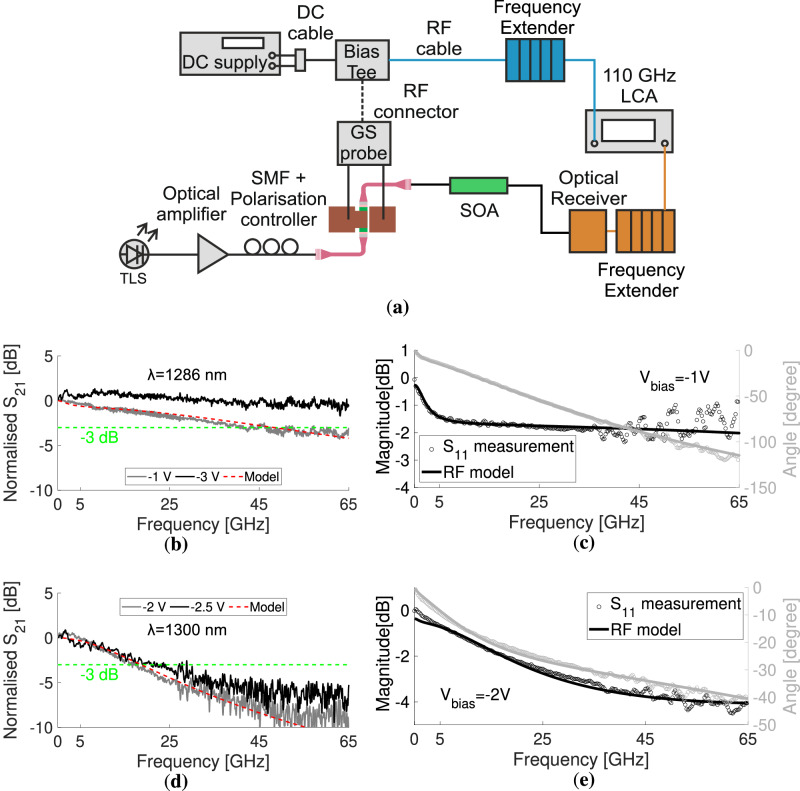
Electro-optical bandwidth measurement. **a** The experimental setup. The *S*_21_ and *S*_11_ response for the Si substrate modulator are given in (**b**) and (**c**), respectively. The corresponding measurements for the SOI scenario are shown in (**d**, **e**). The wavelength of the input signal was 1286 nm and 1300 nm for the Si and SOI substrates, respectively. DC direct current, RF radio frequency, GS ground-signal, SMF single-mode fibre, TLS tunable laser source, SOA semiconductor optical amplifier, LCA lightwave component analyzer.

The electro-optical performance of the modulators was also characterised by measuring their 3-dB bandwidth. A 110 GHz lightwave component analyzer (LCA) was used as shown in Fig. 5, which describes the experimental setup used. The RF system was calibrated before the measurements to decouple them from the electrical effects induced by the setup. The input optical power to the modulators was  ~0.7 mW for the Si and  ~0.43 mW for the SOI. The normalised S_21_ electro-optical response of the Si and SOI substrate modulators is given in Fig. 5b, d, respectively. The measurements were done with frequencies up to 65 GHz because of a 65 GHz limited bias tee and a 67 GHz ground-signal (GS) probe. The responses show a 49 GHz and  >65 GHz 3-dB bandwidth operation for bias values of −1 V and −3 V for the Si substrate. For the SOI substrate, the speeds of 19 GHz and 24 GHz are recorded for −2 V and −2.5 V, respectively. These values are in line with the eye-diagram measurements and further confirm the high-speed capability of the demonstrated waveguide-coupled QCSE modulators. The  >65 GHz speed in combination with the achieved 100 Gb s^−1^ eye-diagram renders the demonstrated modulator as the fastest reported to date.

### Energy consumption

**Fig. 6:**
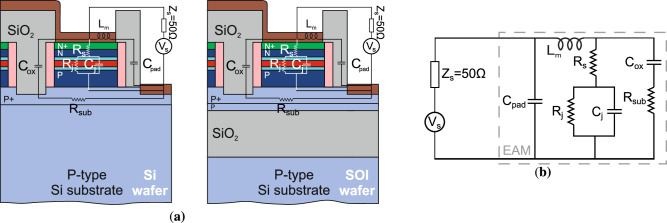
Radio frequency (RF) study of the modulating stacks. **a** The physical depiction on the modulator stack for the Si and SOI wafers and (**b**) the RF equivalent circuit of the electro-absorption modulator.

RF representations of the modulator are illustrated in Fig. 6a, b, following a conventional EAM circuit model as detailed in ref. ^35^. The EAM’s equivalent circuit includes a series resistance *R*_s_, a junction resistance *R*_j_, and a junction capacitance *C*_j_. The series resistance *R*_s_ encompasses both the ohmic contact resistance of the electrodes and the bulk resistance of the doped P+ Si and N+ Si_0.8_Ge_0.2_ contact layers. The junction resistance *R*_j_ is associated with the variable resistance in the MQW region, dependent on the photocurrent. Similarly, the junction capacitance *C*_j_ is linked to the MQW region. Given that *C*_j_ is typically in the femtofarad (fF) range, parasitic capacitance parameters *C*_pad_ and *C*_ox_, accounting for capacitance variations along different metallic pad pathways, were integrated into the model from ref. ^35^, as they are of a comparable magnitude to *C*_j_. Additionally, the model includes the inductance of the metal lines *L*_*m*_ and the substrate resistance parameter *R*_sub_. The RF source in this model is designed with an internal impedance of 50 *Ω*.

Preliminary estimations of the aforementioned parameters were undertaken. Specifically, *R*_s_ can be derived from the slope of the I-V curve’s forward-bias section under high current conditions and absence of illumination. Conversely, *R*_j_ can be inferred from the slope of the I-V curve under reverse bias (current in the range of tens of μA), assuming that the dark current is negligible (current in the range of tens of nA). It is anticipated that *R*_j_ will decrease with increasing input optical power, as the photocurrent rises under constant bias and before optical power saturation is reached. Additionally, *C*_j_ can be estimated using the parallel-plate capacitor formula ${C}_{{{{\rm{j}}}}}=\frac{{\epsilon }_{{{{\rm{r}}}}}{\epsilon }_{{{{\rm{0}}}}}A}{d}$, where *ϵ*_r_ is the relative permittivity of the MQW region, *ϵ*_0_ represents vacuum permittivity, *A* = *W* × *L* is the modulator’s area, and *d* is the MQW region’s thickness, including the spacer layers. The relative permittivity was determined using a weighted fit between the values for undoped Si (*ϵ*_r_ = 11.9) and Ge (*ϵ*_r _= 16). Based on these calculations, the EAM circuit parameters were estimated to be *R*_j_ = 3.42*E*5 *Ω* (with an input optical power of 17.46 μW at 1290 nm) and *R*_s_ = 60 *Ω* for the Si substrate, and *R*_j_ = 1.57*E*5 *Ω* (with an input optical power of 5.6 μW at 1300 nm) and *R*_s_ = 160 *Ω* for the SOI substrate. For both substrates, the junction capacitance related to the EAM geometry is approximated as *C*_j_ = 77.48 fF.

These parameters were derived by fitting the model described above to the electrical response (S_11_) of the device. The bias used for these S_11_ curves were −1 V and −2 V for the Si and SOI cases, respectively, close to the bias of the measured eye diagrams. Figure 5c, e displays the fit for magnitude and phase for the devices on Si and SOI substrates, respectively. Measurements of the electrical response were taken with illumination. Other EAM circuit parameters should remain relatively constant^35^, as they are not significantly affected by photocurrent variations. The fitting process for the Si wafer yielded the following values: *C*_ox_ = 106 fF, *R*_j_ = 3.2*E*4 *Ω*, *R*_s_ = 9 *Ω*, *C*_pad_ = 14 fF, *R*_sub _= 600 *Ω*, *C*_j_ = 49 fF and *L*_m_ = 27 pH. For the SOI device, the values are *C*_ox_ = 30 fF, *R*_j_ = 2.2*E*4 *Ω*, *R*_s_  = 180 *Ω*, *C*_pad_ = 13 fF, *R*_sub _= 4800 *Ω*, *C*_j_ = 40 fF, and *L*_m_ = 3 pH. These fitted values are in line with the initial estimations. Any minor discrepancies in junction capacitance are attributed to unintentional doping affecting the dielectric permittivity, a smaller effective area *A*, and a larger effective distance *d* due to electron/hole localisation at the edges and outer layers of the MQW region. It noted that the higher series resistance of the SOI substrate modulator than the Si case enhances its RC constant. Hence, it explains the lower data rate and bandwidth measured, as compared to the Si substrate modulator. The above fitted circuit parameters were used in the modelling of the *S*_21_ response of the modulator, as can be seen in Fig. 5c, e, for the Si and SOI cases, respectively.

Given the capacitance values and the voltage swings of *V*_DD,Si_ = 2 V for the Si wafer and *V*_DD,SOI_ = 2.5 V for the SOI wafer, the average energy consumption per bit was calculated using the formula $\Delta {E}_{{{{\rm{bit}}}}}=(1/4){C}_{{{{\rm{j}}}}}{{V}_{{{{\rm{DD}}}}}}^{2}$ from ref. ^36^. The results were *ΔE*_bit,Si_ = 49 fJ bit^−1^ and *ΔE*_bit,SOI_ = 62.5 fJ bit^−1^ for the Si and SOI wafers, respectively.

### Thermal performance

To assess the EAM’s performance stability, tests were conducted across a broad temperature range from 20 to 80 °C, mirroring typical data centre conditions^37^. The static extinction ratio for both Si and SOI-substrate modulators was evaluated at various temperatures, regulated using a Peltier heating module.

**Fig. 7:**
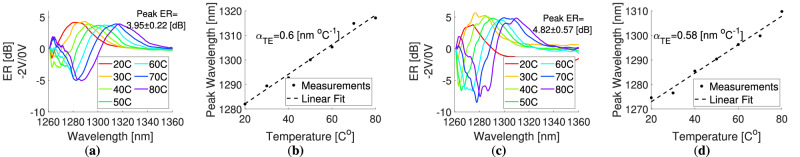
Static performance under temperature variation. Extinction ratio (ER) of the Si and SOI-substrate modulators for the −2 V/0 V bias pair and a 20 to 80 °C temperature range in (**a**) and (**c**), respectively. The drift of the peak wavelength with temperature is shown for the Si and SOI cases at (**b**) and (**d**), respectively.

The extinction ratios for the −2 V/0 V bias pair in Si and SOI configurations are displayed in Fig. 7a, c, respectively. This bias pair was selected for its superior ER performance compared to other pairs, and the figures include devices with the best measured responses. At higher temperatures, the extinction ratio undergoes a red shift due to the band edge shift in the MQW active region towards longer wavelengths as temperature increases^38^. The peak ER values remain relatively stable with temperature increases (Si: 3.95 ± 0.22 dB, SOI: 4.82 ± 0.57 dB), with little variation in peak width. Furthermore, the peak wavelength experiences a drift at similar rates of 0.6 nm °C^−1^ for Si and 0.58 nm °C^−1^ for SOI, as illustrated in Fig. 7b, d, respectively.

To underscore the EAM’s thermal resilience, the peak extinction ratio at a moderate 45 °C was compared with the ERs at 40 °C and 50 °C, measured at the peak wavelength for 45 °C. The Si case showed ER values of 3.55 dB, 4.13 dB, and 3.61 dB, while the SOI device exhibited values of 4.96 dB, 5.09 dB, and 4.46 dB. In both instances, the ER remained above 3 dB within a 10 °C range, with a maximum drop in ER of less than 0.65 dB for both cases.

## Discussion

We developed an 8-in. wafer fabrication process that seamlessly integrates electro-absorption modulators, based on Si-Ge MQW stacks, with SiN waveguides for O-band operation. This approach facilitates a smooth transition between quantum well modulating stacks and silicon nitride waveguides on both Si and SOI substrates. The quality of these active-to-passive interfaces is evident in the achievement of coupling losses below 1 dB and contributes to the high-speed functionality of the modulators, reaching speeds of 100 Gb s^−1^ and  >65 GHz. This rate positions our O-band CMOS-compatible QCSE integrated modulator as the fastest reported to date, providing a pathway to meet the stringent requirements of data communication applications. Table 1 compares our work with the current state-of-the-art monolithic waveguide-integrated QCSE modulators on silicon.

**Table 1:** Performance comparison of monolithic waveguide-integrated QCSE modulators on a silicon substrate

Reference	*λ*[nm]	CL[dB]	BR[dB]	IL[dB]	StaticER [dB]	DynamicER [dB]	Bias[V]	Swing[V]	Data Rate [Gb s^−1^]	Bandwidth[GHz]	C[fF]	*ΔE*_*bit*_[fJ bit^−1^]	Passivewaveguide	W[μm] × L[μm]	LPP[dB]
Thiswork	~1290	1.69 ± 0.49	< −13.58 (O-band)	9.57	3.55 ± 0.22	3.01, 2.28	−1.1, −1	2	50, 100	49 (−1 V), >65 (−3 V) (1286 nm)	49	49	1127 nm N-rich SiN (Si wafer)	2.5 × 50	17.5
Thiswork	~1300	2.08 ± 0.1	< −13.58 (O-band)	10.31	>2.8 ± 1.59	2.74	− 2.3	2.5	50	19 (−2 V) 24 (−2.5 V)	40	62.5	1074 nm N-rich SiN (SOI wafer)	2.5 × 50	~17
^19^	1350, 1465	5, 4	-	16, 15	8, 7	-	-1	1, 2	-	-	-	-	220 nm SOI	1.5 × 40, 1.5 × 80	-
^20^	1321	-	-	~7.5	~3.6	2.66, 2.58, 2.5	-1.5	2	40, 50, 60	50	-	-	220 nm SOI	2 × 40	~12.8
^21^	1340	-	-	8-9	~ 5	-	-2	2	-	> 50	38	38	220 nm SOI	1.25 × 36.8	-

CL coupling loss, *BR* back reflection, *IL* insertion loss, *ER* extinction ratio, *LPP* link power penalty.

Our process leverages the low-temperature growth of PECVD SiN and its defect-free micrometre-scale layering, paving the way for a pilot-line process for fully integrated QCSE MQW modulators on 300 mm diameter wafers. The flexibility of SiN adaptation to various thicknesses of the modulating stack allows for different numbers of quantum wells and buffer layer thicknesses. This method overcomes challenges related to QW quality at the trenches boundary, such as layer bending, thickness variation, and polycrystalline material formation. By using our proposed technique, the uniformity of the electric field within the QWs near interfaces is maintained, and material defects that could increase back-reflections are minimised. This results in lower coupling losses, easing of the link budget constraints, and supports current high-speed modulation requirements of data centres with capabilities of 100 Gb s^−1^.

Additionally, SiN can serve as a connector to standard 220 nm SOI waveguides through evanescent coupling, allowing dual-platform operations. This enables the utilisation of the best components from each material platform for specific applications, such as tight bendings in SOI and temperature-insensitive SiN (DE)MUX. This approach facilitates large-scale photonic integration on SOI wafers, but the process is also applicable to Si substrates, opening the door for cost-effective CMOS electronic-photonic integration on a large scale, with improved heat efficiency.

## Materials and methods

The fabrication process flow for the waveguide-integrated modulators is described below, along with the electro-optical characterisation methods. An illustration of the fabrication process steps can be found in the Supplementary Fig. 3.

### Multiple quantum well stack

The Ge/SiGe MQWs were grown by reduced-pressure chemical vapour deposition at 450 °C on 8-in. <100> Si and SOI P-type substrates with resistivity 18-20 $\Omega \dot{{{{\rm{cm}}}}}$ and 8.5–18 $\Omega \dot{{{{\rm{cm}}}}}$, respectively. The stacks at the two substrate types were grown under identical conditions. However, the BOX and the SOI layers for the SOI case are expected to alter the temperature profile during the growth compared to the Si case. Hence, non-identical stress levels are expected at the buffer layers and the active regions of the two substrates, causing a shift in their peak absorption wavelengths. It is noted that the thicknesses mentioned below refer to the nominal values. The SOI wafer had a 2000 nm BOX layer and a 170 nm P-type SOI epilayer. Two buffer layers were grown on top of the substrates, targeting a 400 nm boron-doped (>1 × 10^19^ cm^−3^) Si layer, followed by a 400 nm boron-doped (~1 × 10^18^ cm^−3^) Si_0.18_Ge_0.82_ layer. The active region was comprised of 8 pairs of Si_0.03_Ge_0.97_ well (~10 nm) and Si_0.33_Ge_0.67_ barriers (~12 nm) layers. Two 15 nm Si_0.18_Ge_0.82_ spacer layers enclosed the active region, followed by phosphorus-doped (~1 × 10^18^ cm^−3^) Si_0.18_Ge_0.82_ and (>1 × 10^19^ cm^−3^) Si_0.8_Ge_0.2_ top contact layers of 300 nm and 82 nm, respectively.

### Active-to-passive interface

For the waveguide interconnection, an initial SiO_2_ layer (200 nm) was deposited through PECVD, acting as a hard mask for the Si-Ge section formation. The Si-Ge mesa (waveguide ridge) was defined by 248 nm DUV lithography through a 0.68 μm thick M91Y resist and the SiO2 hard mask via ICP etching down to a depth of  ~1.5 μm. The Si-Ge sidewalls were passivated by a Si-rich SiN layer grown by PECVD, and the thickness of the layer was tuned so as to act also as the first layer of the double-layer anti-reflective coating (DLARC). The extra material on top of the Si-Ge contact layer and inside the cavities was removed through mask-less ICP etching.

The Si-Ge cavities were then filled with PECVD TEOS (2500 nm). The excessive TEOS material outside the trenches was removed through CMP, forming a flat TEOS layer inside the trenches. The bottom cladding of the passive SiN section (thickness controlled by etching) and the second DLARC layer (length controlled by lithography) were defined by 248 nm DUV lithography using a 1.3 μm thick M91Y resist and ICP etching down to 1100 nm on an in-cavity flat TEOS surface.

### SiN waveguides

Following the TEOS ICP etching, the N-rich SiN was deposited at 350 °C as described in ref. ^24^, and planarised via CMP down to the thickness of  ~1100 nm. After the planarisation step, the rib N-rich SiN waveguides of a 368 nm thick slab were formed through 248 nm DUV lithography using 1 μm M91Y resist and ICP etching. The waveguides were capped by PECVD SiO_2_, with a thickness of 750 nm.

### Integrated QCSE modulators

The formation of the vias for the N and P contact layers followed, through 248 nm DUV lithographies and RIE etching of the SiO_2_ down to a thickness of 50 nm. Further wet etching with hydrofluoric acid removed the thin oxide layer and any native oxides on the top N-type Si_0.8_Ge_0.2_ and bottom P-type Si_0.18_Ge_0.82_ contact layers. The bottom and the top metals were sputtered sequentially, composed of a 30 nm/24 nm/220 nm/10 nm/16 nm Ti/TiN/Al/TiN/Ti metal stack. Finally, the metal pads were defined through lift-off.

### Optical measurements

The devices were characterised optically using an Agilent 8164B tunable laser source with a wavelength tuning range between 1260 nm and 1360 nm. The polarisation of the light was controlled to achieve the TE excitation of the modes. Single-mode fibres were used for the fibre-to-chip interconnection through grating couplers with straight gratings realised on the N-rich SiN waveguides. The gratings had a width of 10 μm, a period of 1016 nm, a filling factor of 50%, and 30 teeth in total. The N-rich SiN waveguide was narrowed down from 10 μm to its single-mode width of 1.1 μm through a 510 μm long adiabatic taper for the adiabatic transition of the fundamental propagating mode. A 90° bending of 140 μm radius followed for further elimination of any potential high-order modes excited at the fibre-grating coupler connection. Subsequently, a 130 μm long adiabatic taper was used for the transition of the single-mode width to the width of the modulator, leading without losses only the fundamental mode at the interface. The width of the modulator allows the existence of high-order modes, which can propagate bidirectionally and independently along with the fundamental. The device is anti-symmetric in the propagation direction, so the same mode transitioning and filtering scheme of the input section is employed at the output section. Consequently, as for the forward propagation, any high-order modes excited at the interface were filtered, and only the fundamental mode was guided adiabatically to the output grating coupler. Also, any back-reflection from high-order modes was leaked out through the filtering mechanism described above. In this way, the excitement, propagation, modulation, and measurement at the output fibre of the fundamental mode is guaranteed.

### Electrical and high-speed measurements

The setup used for the eye-diagram measurement is presented in Fig. 4a. The high-speed electrical pulse was generated by using two electrical signals (pseudorandom binary sequence (PRBS) 2^11^–1) that were generated by an SHF bit-pattern generator (12104A) and were sent to an SHF 603B MUX. The MUX provided a seed signal at data rates up to 100 Gb s^−1^. The clocking was achieved by an Agilent E8257D clock generator. To apply the seed signal on the modulator, a 0.5 m 67 GHz cable was used to connect the MUX output with a 60 GHz amplifier (SHF S804), followed by a 65 GHz bias tee and finally a 50 *Ω*-terminated 67 GHz GGB GS probe. The modulated output light was amplified by connecting the output fibre to an O-band amplifier (FiberLabs AMP-FL8611-OB 1280–1320 nm). The optical noise of the amplified light was reduced through a bandpass filter and it was fed into a Keysight digital communication analyser (Keysight Infiniium DCA-X 86100D with an Agilent 86116C-040 plugin module). The electrical signal tuning was implemented by the software functions of DCA. The eye diagrams were measured with pattern locking at the corresponding data rate and with averaging, using also a six-tap FFE for a better depiction of the eye diagrams.

The EO bandwidth (S_21_ parameter) and the electrical response (S_11_ parameter) under light coupling, were measured with the setup shown in Fig. 5a. Specifically, a lightwave component analyzer (LCA) was used (Keysight N4372E) with a performance network analyzer (PNA) millimetre test set (Keysight, N5292A). The RF electrical signal was guided from the PNA to a frequency extender (FE, Keysight N5293AX01 10 MHz–110 GHz), which was connected to a 65 GHz bias tee through a 0.5 m 67 GHz cable, followed by a 67 GHz GGB GS probe. The amplifier of the eye-diagram setup was used at the optical input of the modulator. Also, the output light was amplified by connecting the output fibre to an O-band semiconductor optical amplifier (SOA, Maiman Electronics SF8075). The light was received by an LCA optical receiver (N4372-67985 110 GHz) and fed into the PNA by another frequency extender. The fit of the magnitude in dB and the angle in degrees, along with the *S*_21_ modelling, was done using the commercial *Advanced System Design 2008* software. For the temperature-controlled measurements, the chips were put on a Peltier-equipped stage, which was operated by a temperature controller (Thorlabs, TED200C).

## Supplementary information

Supplementary Information

## Data Availability

The data underlying the results presented in this paper are not publicly available at this time, but may be obtained from the authors upon reasonable request.
