# Supplementary material for: A 100 Gb s−1 quantum-confined Stark effect modulator monolithically integrated with silicon nitride on Si
Source: Commun Eng. 2025 May 1;4:82. doi: 10.1038/s44172-025-00421-6 (PMC12046052; doi:10.1038/s44172-025-00421-6)
Supplement: Supplementary file 1 — Supplementary Information [file 44172_2025_421_MOESM1_ESM.pdf]

Supplementary Information: *A 100 Gb s^{-1} quantum-confined Stark effect modulator monolithically integrated with silicon nitride on Si*

Ilias Skandalos[1,*], Thalía Domínguez Bucio[1], Lorenzo Mastronardi[1], Guomin Yu[2], Aaron Zilkie[2], and Frederic Y. Gardes[1]

[1]Optoelectronics Research Centre, University of Southampton, Southampton, SO17 1BJ, UK
[2]Rockley Photonics, Inc., Pasadena, CA 91104, USA
[*]I.Skandalos@soton.ac.uk

Active-to-Passive Coupling

In this study, a N-rich silicon nitride (SiN) composition with a refractive index of $n = 1.9$ was utilised as the foundational material for the passive waveguide platform. This choice was motivated by its demonstrably low propagation losses in the O-band, ranging from 1 to 2 dB cm^{-1}[1]. Furthermore, this material presents advantageous fabrication considerations for applications in coarse wavelength division multiplexing (CWDM)[2], while it can also offer wavelength trimming capabilities for resonant structures in a post-fabrication manner[3]. A robust optical modulation necessitates a low-loss and low-reflection interface between the active silicon-germanium (Si-Ge) and the passive SiN waveguides. To address this requirement, a butt-coupling technique was employed, thoroughly described by Skandalos et. al. (2022) in Supplementary Ref. 4. This coupling strategy is critical in managing the losses and reflections at the juncture of the active and passive waveguides. The fabrication process mitigates the drawbacks typically associated with the integration of active and passive waveguides, by embedding the amorphous passive waveguide layers within the Si-Ge cavities, instead of the conventional method of growing Si-Ge layers within a SOI cavity. This method effectively prevents the formation of defective Si-Ge layers at the boundary, leading to increased optical losses, reflections as well as modulation uncertainty due to non uniformity in the MQW stack. Consequently, this approach eliminates the need for processing of defective material from the interfaces. Instead, chemical-mechanical polishing (CMP) steps are implemented to enable the formation of amorphous waveguiding layers. This substitution ensures the precise control over the structure, position, thickness, and length of the waveguides and the layers at the interfaces, enhancing the overall reliability and performance of the active waveguiding system.

High coupling losses between the waveguides, attributed to discrepancies in the modal spatial alignment and the effective index differences can limit the overall link budget[5]. Additionally, the possibility of parasitic back-reflections, due to these modal mismatches, can also lead to destructive interference with the modulated mode, resulting in undesirable spectral dips that negatively impact the modulation quality. To address these challenges, the geometry of the N-rich SiN waveguide was fine-tuned to achieve better spatial alignment with the optical mode of the Si-Ge waveguide. Furthermore, a double-layer anti-reflective coating (DLARC), comprising tetraethyl orthosilicate (TEOS) with a refractive index of $n = 1.44$ and a silicon-rich SiN layer with a refractive index of $n \sim 2.5$, was employed to reduce back-reflections. Supplementary Fig. 1 provides a visual representation of the waveguides' cross-sections and illustrates a two-dimensional view of the transition from the active to the passive waveguides.

Supplementary Fig. 1(**a**) illustrates the cross-sectional views of the N-rich SiN waveguide, the DLARC composed of silicon-rich SiN and TEOS layers, and the Si-Ge waveguide, in relation to the SOI wafer. Supplementary Fig. 1(**b**) presents a side view of the structure along the mode propagation direction, indicated by the PP' arrow, for Si and SOI wafers. It should be noted that the interface between the waveguides is flat. In this structure, the N-rich SiN section is designed as a rib waveguide. The DLARC portion comprises the TEOS and silicon-rich SiN slab layers, while the Si-Ge segment is constructed as a ridge waveguide. The structures are encapsulated in SiO_2 top and bottom claddings of thicknesses t_{TOX} and t_{BOX}, respectively. The bottom cladding for the N-rich SiN waveguide has an extra TEOS layer of thickness $t_{\mathrm{BOX,TEOS}}$ to allow for correct positioning in reference with the Si-Ge waveguide. The fundamental TE modes for the SiN and the Si-Ge waveguides are shown in the Supplementary Fig. 1(**c**) and Supplementary Fig. 1(**d**), respectively.

The waveguides' alignment along the growth direction was meticulously engineered through 3D finite-difference time-domain (3D-FDTD) simulations, employing S-parameters to achieve optimal mode matching for the fundamental transverse electric (TE) polarisation. The SiN core (t_{core}) and slab (t_{slab}) thicknesses, along with the widths of the SiN (w_{SiN}) and the Si-Ge ($w_{\mathrm{Si\text{-}Ge}}$) waveguides were specifically designed to enhance optical coupling efficiency. Additionally, the Si-Ge waveguide

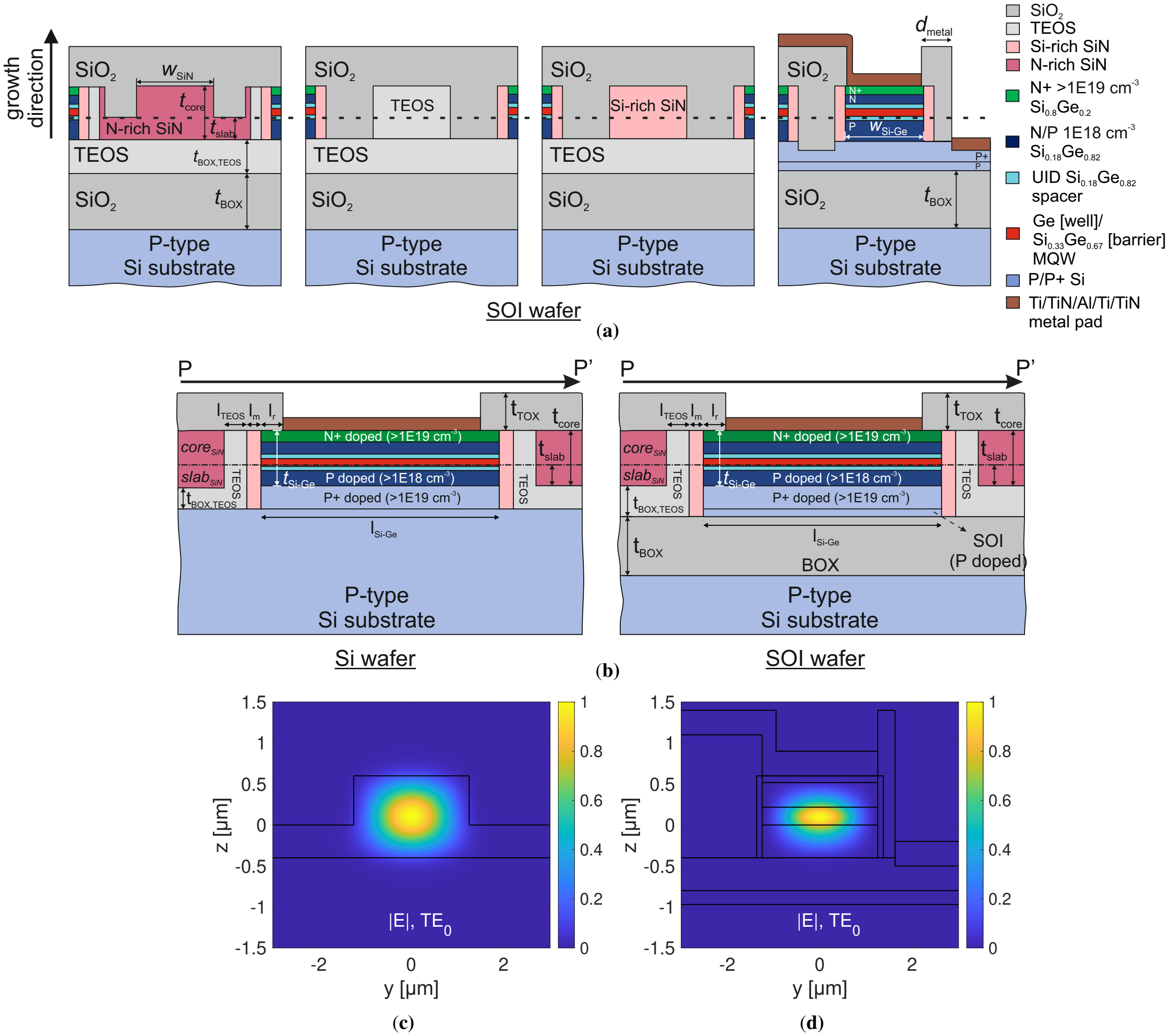

Supplementary Fig. 1. 2D illustration of the modulator integration. (a) The cross-sections of the N-rich SiN and Si-Ge waveguides along with the Si-rich SiN slab ARC layer for the case of an SOI wafer. (b) The active-to-passive transition for both cases of Si and SOI wafers. The electric field intensity of the TE_0 mode for the (c) SiN and (d) Si-Ge waveguides.

width was adjusted to maximise modal overlap with the quantum wells, thereby optimally leveraging the efficiency of the QCSE. The longitudinal TEOS layer, with a length of l_{TEOS}, is crucial for compensating the overlay precision of less than 20 nm, required for the 248 nm DUV lithography employed in this work. Without this layer, the thickness l_{m} of the Si-rich layer could be compromised during the dry etch process used for defining the buried oxide (BOX) cladding, potentially disrupting the anti-reflective properties of a sensitive single-layer anti-reflective coating (ARC), in addition to a physical distortion on the layer that would adversely affect the propagating mode. The introduction of the TEOS layer effectively mitigates issues related to lithographic overlaps and also aids in maintaining high-quality coupling by setting optimised lengths for both the TEOS and Si-rich SiN slab layers. Moreover, a recess of length l_{r} was intentionally created for the top metal pad at the interface atop the Si-Ge waveguide. This design choice ensures that the metal pad does not interfere with the weakly confined mode near the interface in the N-rich SiN section. The metal pad is positioned over the Si-Ge stack, which has a thickness of $t_{\text{Si-Ge}}$, in a region where the mode is well-established and confined within the Si-Ge waveguide. Despite this, the electric field is considered to be uniformly applied across the entire Si-Ge waveguide, owing to the high N+ doping concentration (10^{19} cm^{-3}). The two metal pads are separated laterally by a distance of $d_{\text{metal}} = 500\,\text{nm}$.

The butt-coupling scheme between the waveguides was also explored through numerical analysis using a 3D-FDTD simulation on the commercial software *FDTD Solutions* from Lumerical. This study focused on analysing the power coupling loss and the back-reflection. Due to the polarisation sensitivity of the Ge/SiGe MQW stack, as discussed in Supplementary Ref.

6, the investigation was limited to the propagation of the fundamental TE mode across a single interface. Initially, the core and the slab thicknesses of the N-rich SiN waveguide were examined, followed by a series of simulation sweeps assessing the lengths of the TEOS and Si-rich SiN layers, and further parametric studies on the widths of the N-rich SiN and Si-Ge waveguides.

For a comprehensive evaluation of a multi-parameter interconnection system, it is crucial to consider a broad spectrum of material and geometric variables to assess its robustness. This range is influenced by various factors, including variations in the thickness of multiple layers in the active stack (impacting the optimal thickness and positioning of the passive waveguide), lithographic challenges (such as alignment errors and over- or under-exposure), fabrication processes (dimensional changes during etching, refractive index shifts during deposition), and constraints in characterisation techniques (like ellipsometry measurements on multi-layer dielectric stacks). Following the statistical analysis methodology detailed in Supplementary Ref. 4 of Skandalos et al. (2022), a set of values around a designated design simulation point is numerically explored. This exploration focuses on evaluating the coupling loss and the back-reflection. The spread of these metrics across the range of variables in the multi-parameter study, serves as an indicator of the sensitivity and reliability of the coupling mechanism. The distribution of the studied metrics under these ranges is plotted in Supplementary Fig. 2(**a**). The operational wavelength for this statistical analysis was set at 1310 nm. For the statistical spread, a population of 100 uniformly distributed points was used, and a separate propagation simulation was ran for each of them. The design point of 0.72 dB coupling loss and −18.11 dB back-reflection is represented by a cross ("Design" case in Supplementary Table 1), while all the randomly generated combinations are plotted as diamonds ("Range" case in Supplementary Table 1). It is important to note that this design point was selected primarily to optimise the coupling loss, aiming to achieve the lowest possible insertion loss. Given constant parameters for the Si-rich SiN, the optimal point for minimising the back-reflection corresponds to a TEOS length of l_{TEOS} =414 nm, resulting in a back-reflection value of −24.33 dB ("Optimum" case in Supplementary Table 1). At this setting, the coupling loss slightly increases to 0.76 dB. However, there is a potential for further elevation in the coupling loss due to fabrication variations, which may be due to an extended length of the propagation in the TEOS slab. Statistical analysis from the graph suggests that the interface maintains a coupling loss near or even below 1 dB. Simultaneously, back-reflections can be kept around −18 dB. This performance aligns with contemporary laser back-reflection tolerances, which range from −14 dB up to −7 dB, as discussed by Grillot et al. (2020) in Supplementary Ref. 7 and Lu et al. (2022) in Supplementary Ref. 8.

The coupling quality of the interface was investigated experimentally using a cut-back structure. The cut-back structure consisted of metallised Si-Ge waveguides of increasing length (50 µm-600 µm), as shown in Supplementary Fig. 2(**b**). The initial step in the analysis involved calculating the optical insertion loss per unit length for the metallised Si-Ge waveguide, (excluding the interfaces loss) which was measured to be approximately $\sim$ 1440 dB cm^{-1} at 1290 nm and $\sim$ 1266 dB cm^{-1} at 1300 nm. This was done by normalising the optical response across all devices, using the shortest device as a reference. It is important to note that the Si-Ge sections were unbiased, with a 0 V applied. To determine the loss at one interface, the overall measured response was adjusted by deducting the aforementioned Si-Ge insertion loss, the insertion loss of the N-rich SiN passive part (approximately $\sim$2.10 $\pm$ 0.17 dB cm^{-1}), and the loss from the grating couplers. This calculation was done for the wavelength range of 1290 nm-1350 nm, as shown in Supplementary Fig. 2(**c**). Below 1290 nm, the insertion loss for Si-Ge was too high to establish an accurate fit, and above 1350 nm the high insertion loss of the grating couplers impeded precise calculation. The coupling loss values, expressed as mean and standard deviation, were derived from different devices used in the cut-back structure of a single chip from the Si wafer. This particular chip was identified as having the optimal exposure dose in a lithographic dose sweep for the Si-Ge waveguide layer. Similar values were measured for the SOI wafer. The analysis primarily utilised the first four devices (lengths: 50 µm, 150 µm, 250 µm and 400 µm), as the insertion loss in longer devices was excessively high for accurate fitting. According to Supplementary Fig. 2(**b**), the average coupling loss in the 1290 nm-1350 nm range is close to 1 dB. Specifically, at 1290 nm, 1300 nm and 1305 nm, the coupling losses are 1.69 $\pm$ 0.49 dB, 2.08 $\pm$ 0.10 dB and 1.11 $\pm$ 0.47 dB, respectively.

Similar cut-back structures were used to estimate the back-reflection of each interface. Specifically, a series of cascaded interfaces between N-rich SiN and non metallised Si-Ge waveguides were fabricated to investigate the interface reflecting properties, in combination with a non metallised Si-Ge cutback structure used to calculate the propagation loss of the Si-Ge waveguide, as presented in Supplementary Fig. 2(**d**) and 2(**e**), respectively. The Si-Ge waveguides are not metallised in order to avoid extra insertion loss due to the metallic pads, that would potentially add further loss to the Si-Ge waveguide. The N-rich SiN waveguide sections with a length of 210 µm contain a taper section in order to filter out potential higher-order modes, while 200 µm long Si-Ge waveguides were used to avoid enhanced reflections due to potential unsettled mode propagation. The estimated back-reflection is assumed to be very similar to an interface with a metallised Si-Ge section, because of the 2 µm intentional recess that was left on both sides of the Si-Ge waveguide. It is important to note that this structure could be used as a cross-validation of the coupling loss data, however the attenuation of the Si-Ge long sections ($\sim$350 dB cm^{-1} at 1310 nm) was too high for a precise fit. The reflectivity of one interface was calculated based on the fit of the spectral optical measurement to the simulated response using a scattering matrix model, as described in Supplementary Ref. 4. A device with 6

interfaces in total was used to compare its measured and simulated responses near 1310 nm as plotted in Supplementary Fig. 2(f). The wavelength range of 1310 nm-1320 nm was chosen due to the similar level of Si-Ge waveguide propagation loss in these wavelengths. It should be pointed out, that an envelope of the measurement response curve was used, and that the simulation curve was normalised to its maximum value for a fair comparison. Based on the fit, a reflectivity of -13.98 dB at 1310 nm and less than -13.58 dB in the whole O-band is calculated, which falls into the simulated statistical range. These measurements refer to one chip of the SOI wafer that was exposed with the optimal exposure dose in a lithographic dose sweep for the Si-Ge waveguide layer. Similar values were measured for the Si wafer.

Supplementary Table 1. Investigated ranges of values for the geometric and the material parameters related to the active-to-passive interface; the case of a flat interface is investigated.

Case	t_{core} [nm]	t_{slab} [nm]	n_{NSiN}	l_{TEOS} [nm]	l_m [nm]	n_m	l_r [nm]	w_{SiN} [nm]	$w_{Si\text{-}Ge}$ [nm]	t_{TOX} [nm]	$t_{Si\text{-}Ge}$ [nm]
Design	1000	400	1.9	330	120	2.5	2000	2500	2500	750	1000
Optimum	1000	400	1.9	414	120	2.5	2000	2500	2500	750	1000
Range	[800,1200]	[350,450]	[1.88,1.92]	[250,450]	[100,140]	[2.4,2.6]	[1980,2020]	[2450,2550]	[2450,2550]	[700,800]	[750,1250]

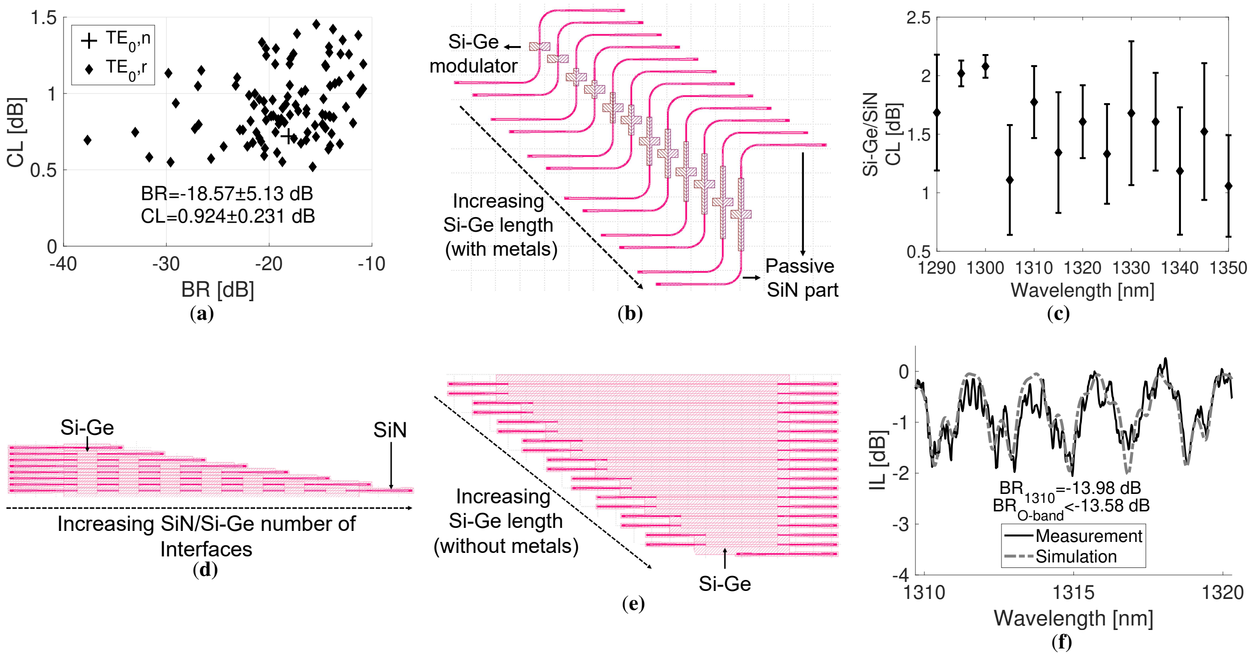

Supplementary Fig. 2. (a) Statistical spread of the simulated coupling loss (CL) and back-reflection (BR) metrics under uniformly varying material and geometric parameters in restricted ranges. (b) A metallised Si-Ge cutback structure for the coupling loss measurement; the measured coupling loss is depicted in (c) for different wavelengths. Cutback structures of (d) not metallised multiple Si-Ge/SiN interface sections and (e) not metallised Si-Ge sections of multiple lengths for the back-reflection measurement; the back-reflection calculation is seen in (f).

The Si-Ge waveguide's thickness was found to be approximately $\sim$243 nm greater than the expected stack thickness of 1000 nm. This increase not only expands the mode diameter in the Si-Ge waveguide but also impacts the relative alignment of the N-rich SiN waveguide. The basis of the N-rich SiN waveguide is aligned with the lower P-doped $Si_{0.18}Ge_{0.82}$ layer of the Si-Ge waveguide. Coupled with the fixed position of the N-rich SiN waveguide, this leads to a misalignment of the centres of the two waveguides from their optimal positions, significantly influencing the coupling loss between them. This misalignment is a primary factor in the divergence observed between the simulated and actual measurements. Regarding back-reflection, it is predominantly influenced by the lengths and refractive indices of the layers in the anti-reflective coating.

Fabrication Process Steps

Supplementary Fig. 3 illustrates the fabrication steps for the modulator integration.

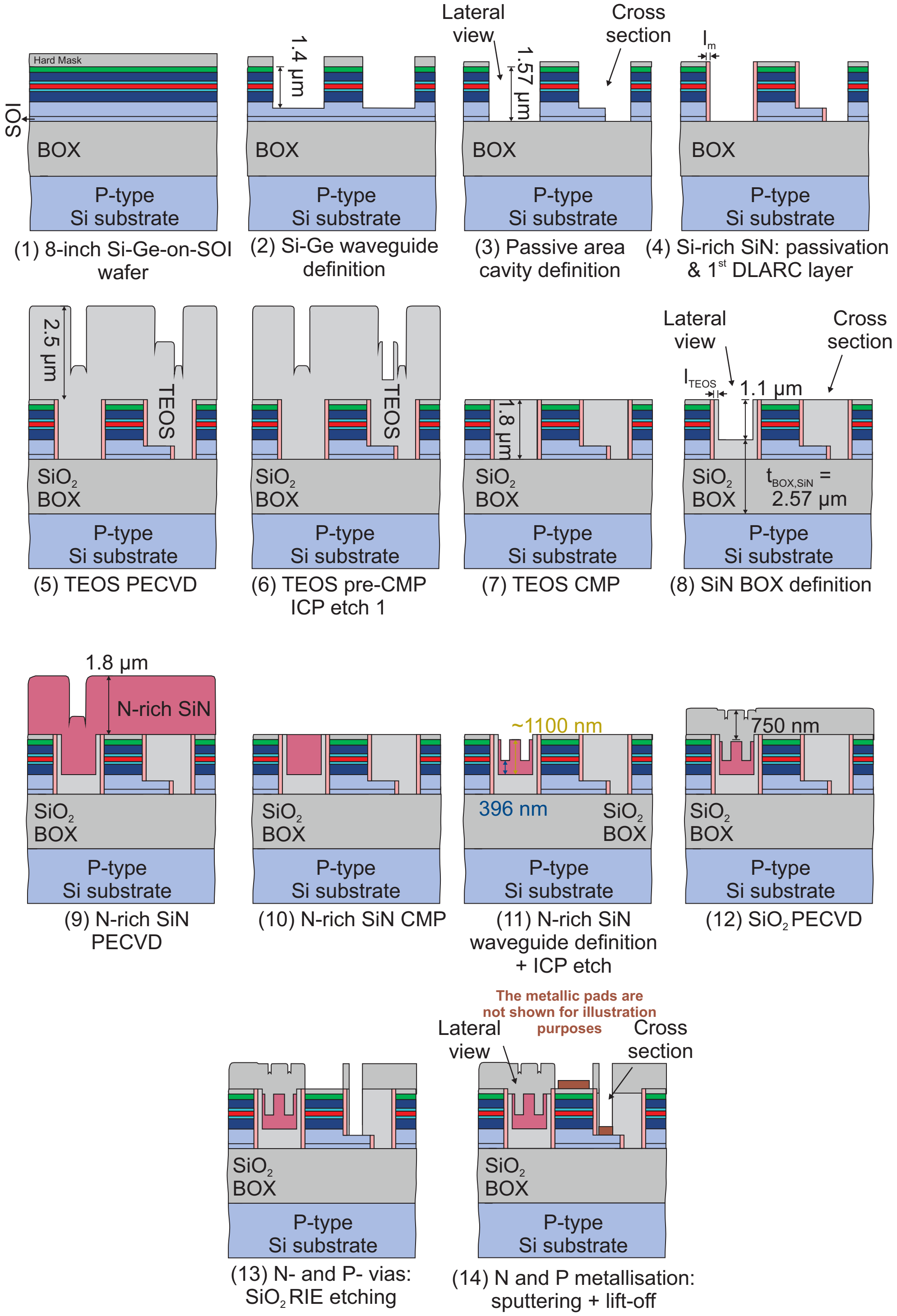

Supplementary Fig. 3. Fabrication process flow. The case of an SOI substrate is shown, while the same process was also followed for the Si substrate.

Supplementary References

1 Bucio, T. D. *et al.* Material and optical properties of low-temperature NH_3-free PECVD SiN_x layers for photonic applications. *J. Phys. D: Appl. Phys.* **50**, 025106 (2017).

2 Bucio, T. D., Khokhar, A. Z., Mashanovich, G. Z. & Gardes, F. Y. N-rich silicon nitride angled MMI for coarse wavelength division (de)multiplexing in the O-band. *Opt. Lett.* **43**, 1251 (2018).

3 De Paoli, G. *et al.* Laser trimming of the operating wavelength of silicon nitride racetrack resonators. *Photonics Res.* **8**, 677 (2020).

4 Skandalos, I., Bucio, T. D., Mastronardi, L., Rutirawut, T. & Gardes, F. Y. Coupling strategy between high-index and mid-index micro-metric waveguides for O-band applications. *Sci. Reports* **12**, 17453 (2022).

5 Heck, M. J. R. & Bowers, J. E. Energy Efficient and Energy Proportional Optical Interconnects for Multi-Core Processors: Driving the Need for On-Chip Sources. *IEEE J. Sel. Top. Quantum Electron.* **20**, 332–343 (2014).

6 Chaisakul, P. *et al.* Polarization dependence of quantum-confined Stark effect in Ge/SiGe quantum well planar waveguides. *Opt. Lett.* **36**, 1794 (2011).

7 Grillot, F. *et al.* Physics and applications of quantum dot lasers for silicon photonics. *Nanophotonics* **9**, 1271–1286 (2020).

8 Lu, Y. *et al.* Analysis of the regimes of feedback effects in quantum dot laser. *J. Phys. D: Appl. Phys.* **55**, 484003 (2022).
